# Supplementary figures and images for: Spatially resolved in silico modeling of NKG2D signaling kinetics suggests a key role of NKG2D and Vav1 Co-clustering in generating natural killer cell activation
Source: PLoS Comput Biol. 2022 May 18;18(5):e1010114. doi: 10.1371/journal.pcbi.1010114 (PMC9154193; doi:10.1371/journal.pcbi.1010114)

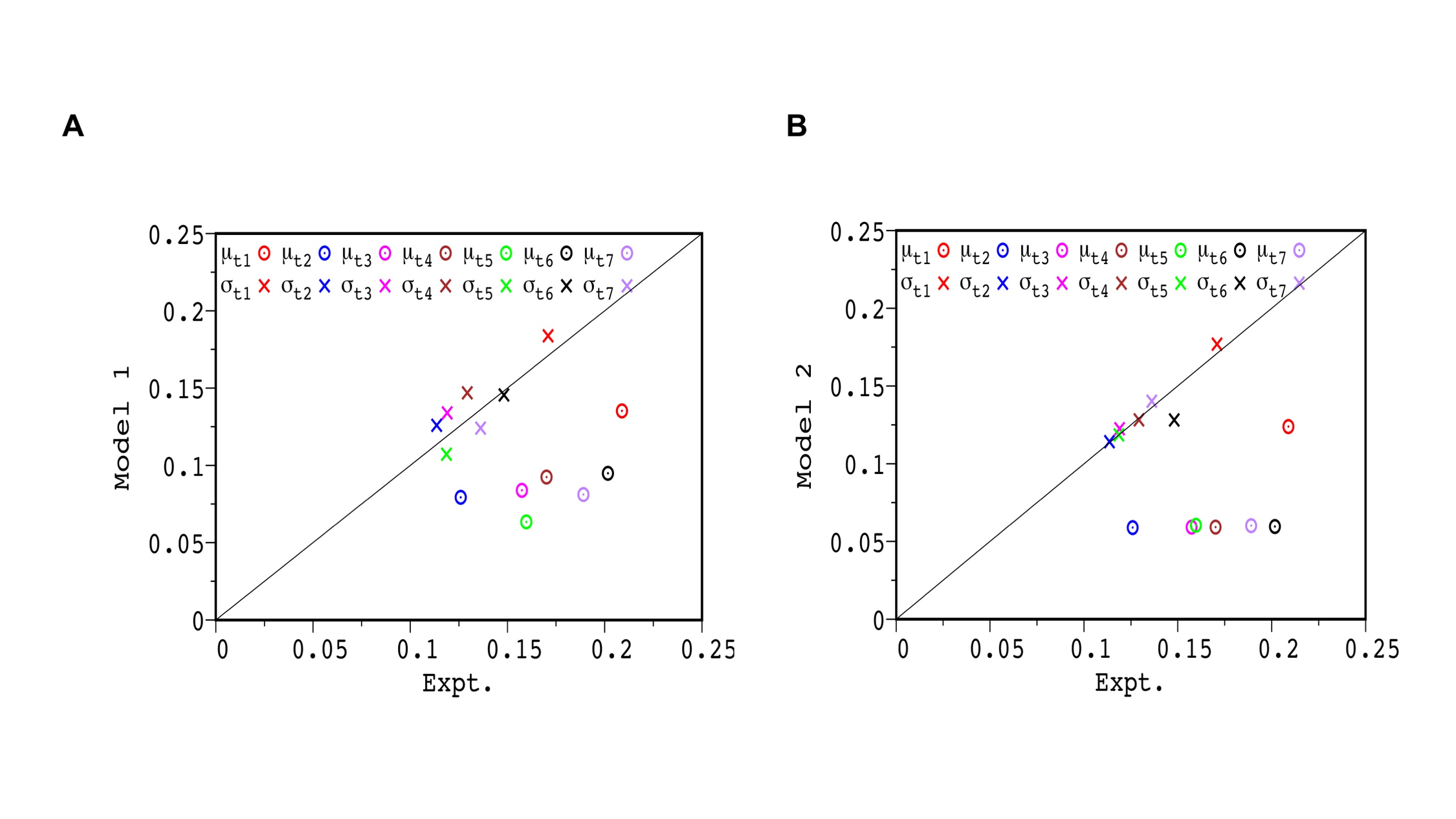

Supplement: S1 Fig — Shows μI and σI calculated from TIRF images (S4 Fig in Ref. [6]) and model simulations at t = 1, 2, 3, 4, 5, 6, and 7 mins. The calculations of μI and σI are shown in Eq 2a and 2b in the main text. The values for TIRF images and model simulations are shown along the y and x axes, respectively. The x = y line is shown to quantify agreement/deviation between imaging data and models. The symbols indicated by μa and σa depict the values of (μI, μn) and (σI, σn) in the x-y plane at times a = t1 to t7 denoting times 1 to 7 mins, respectively. Comparisons are shown for (A) Model 1 and (B) Model 2. The models are simulated for the best-fit PSO parameters. (TIF) [file pcbi.1010114.s001.tif]

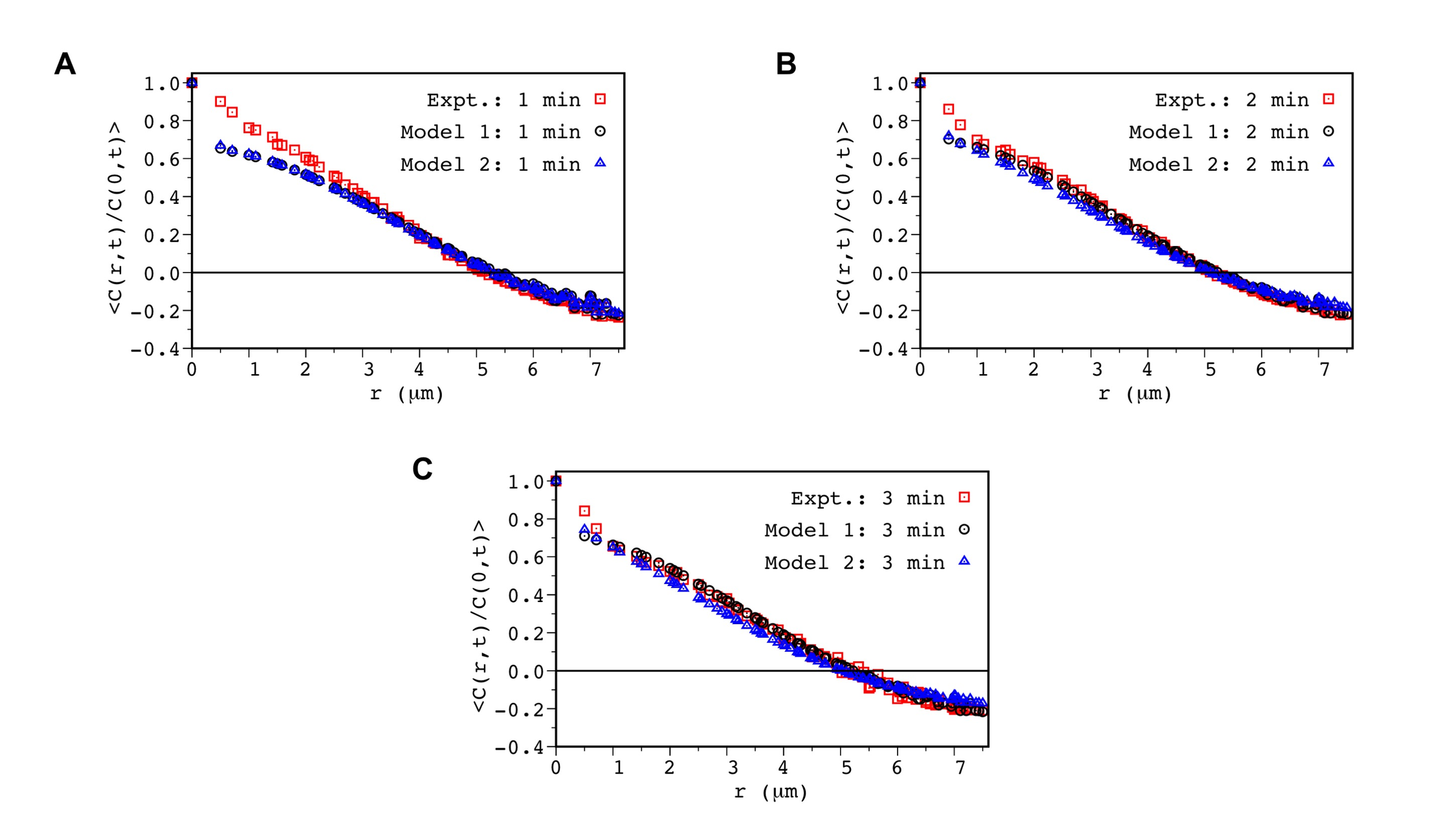

Supplement: S2 Fig — Shows comparisons between ensemble averaged two-point correlation function (<C(r,t)/C(0,t)> vs r) for Model 1 (black, empty circle) and Model 2 (blue, empty triangle) with C(r,t)/C(0,t) calculated from TIRF image (red, empty square) at (A) t = 1min, (B) t = 2 min, and (C) t = 3 min. The parameters for the simulation are set at the best-fit values from the PSO. The two-point correlation functions for the models are averaged over an ensemble of 200 configurations. (TIF) [file pcbi.1010114.s002.tif]

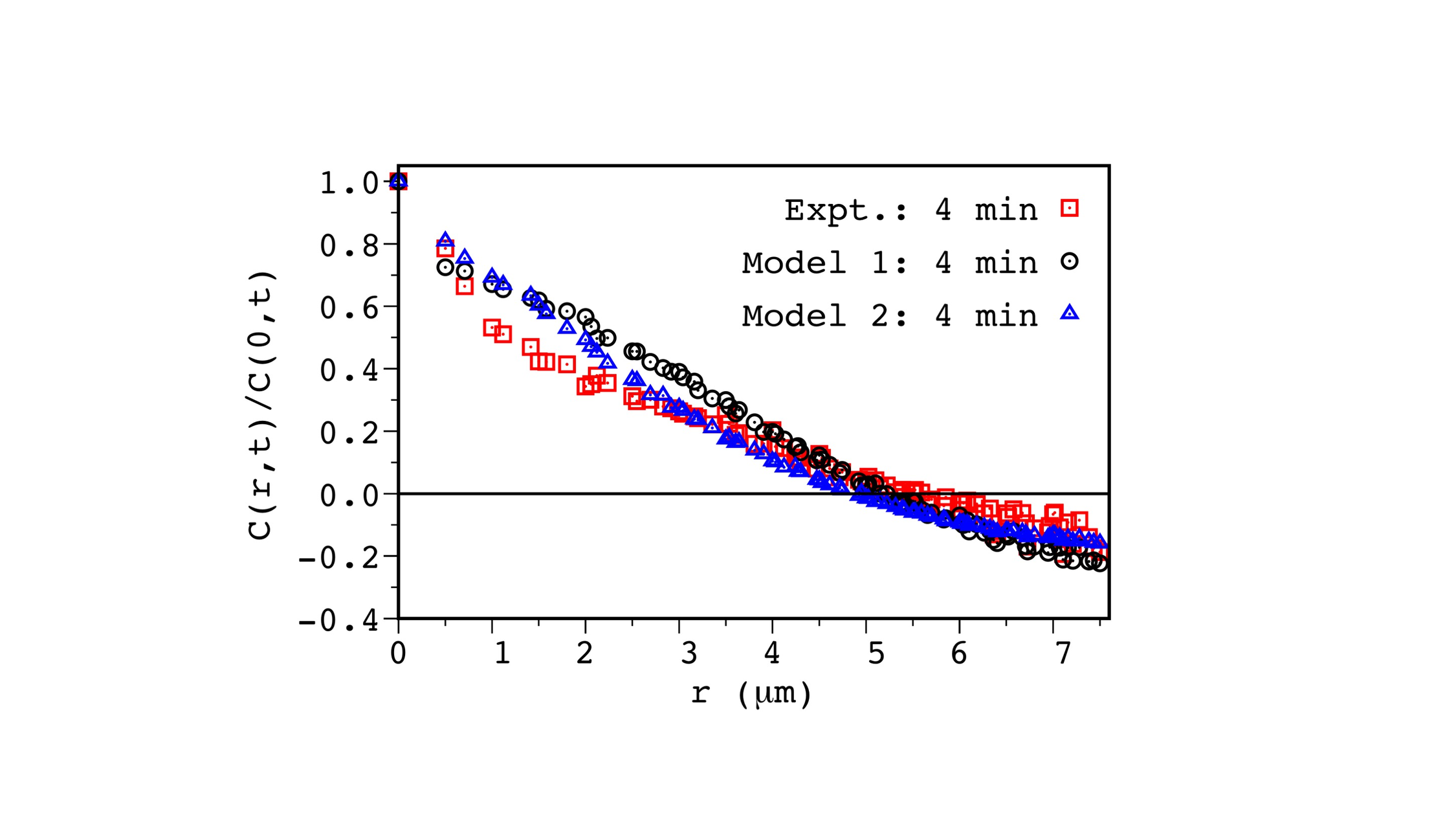

Supplement: S3 Fig — Shows comparison between the two-point correlation function (C(r,t)/C(0,t) vs r) at t = 4 min calculated from TIRF image (red, empty square) and configurations simulated by Model 1 (black, empty circle) and Model 2 (blue, empty triangle). The parameters for the simulations are set to the best-fit values obtained from our PSO. The models show deviations from the TIRF imaging data for length scales 1–3 μm which can potentially arise due to substantial spreading of the NK cell on the lipid bilayer at 4 mins. (TIF) [file pcbi.1010114.s003.tif]

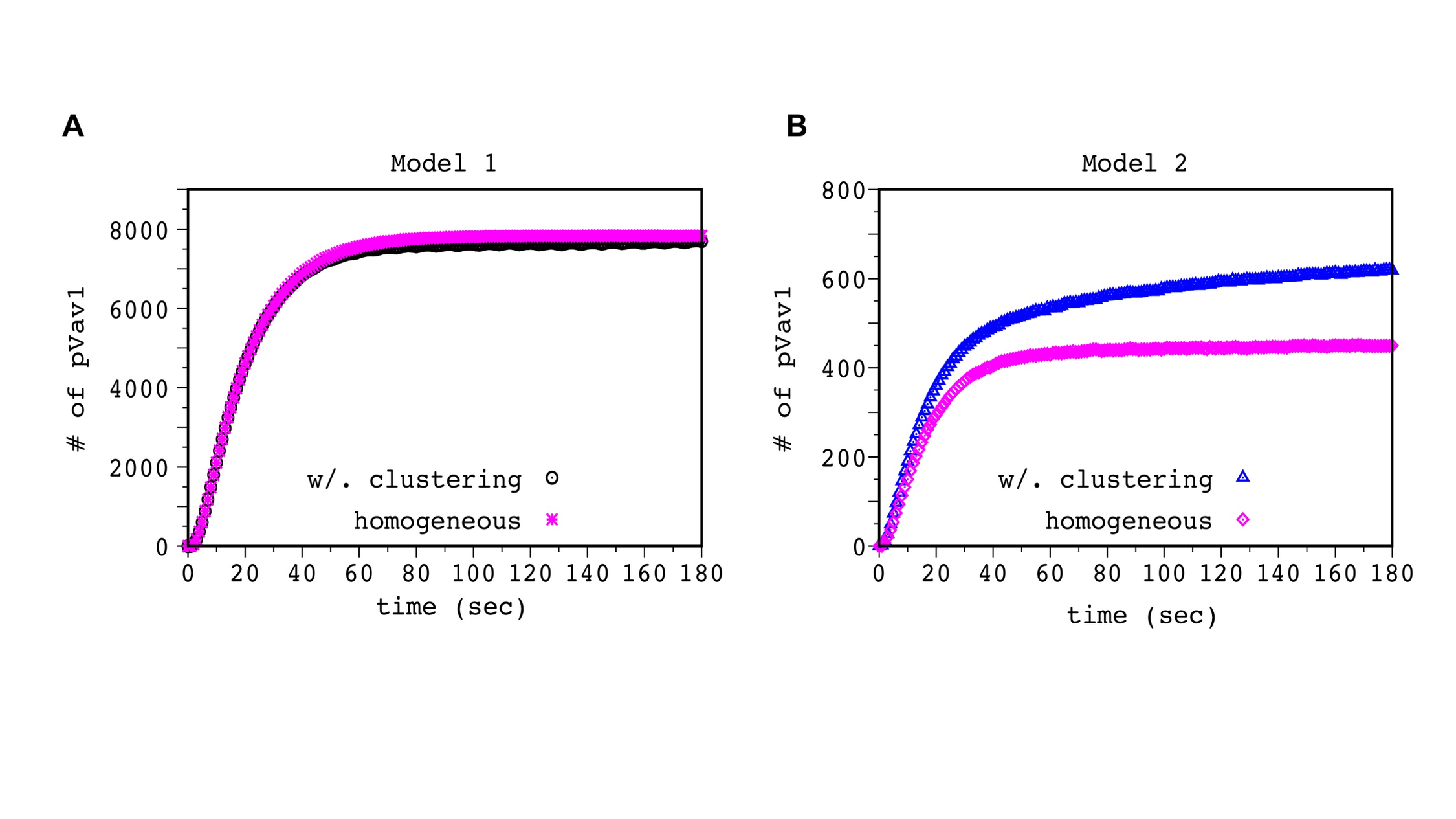

Supplement: S4 Fig — Shows increase in the total number of pVav1 with time in Model 1 (A) and Model 2 (B) when NKG2D are not allowed to form microclusters (magenta asterisks, Model 1; magenta diamonds, Model 2) or form microclusters (black open circles, Model 1; blue open triangles, Model 2) according to the model rules. The pVav1 concentrations are averaged over 200 different configurations. The models are simulated for the best-fit PSO parameters. (TIF) [file pcbi.1010114.s004.tif]

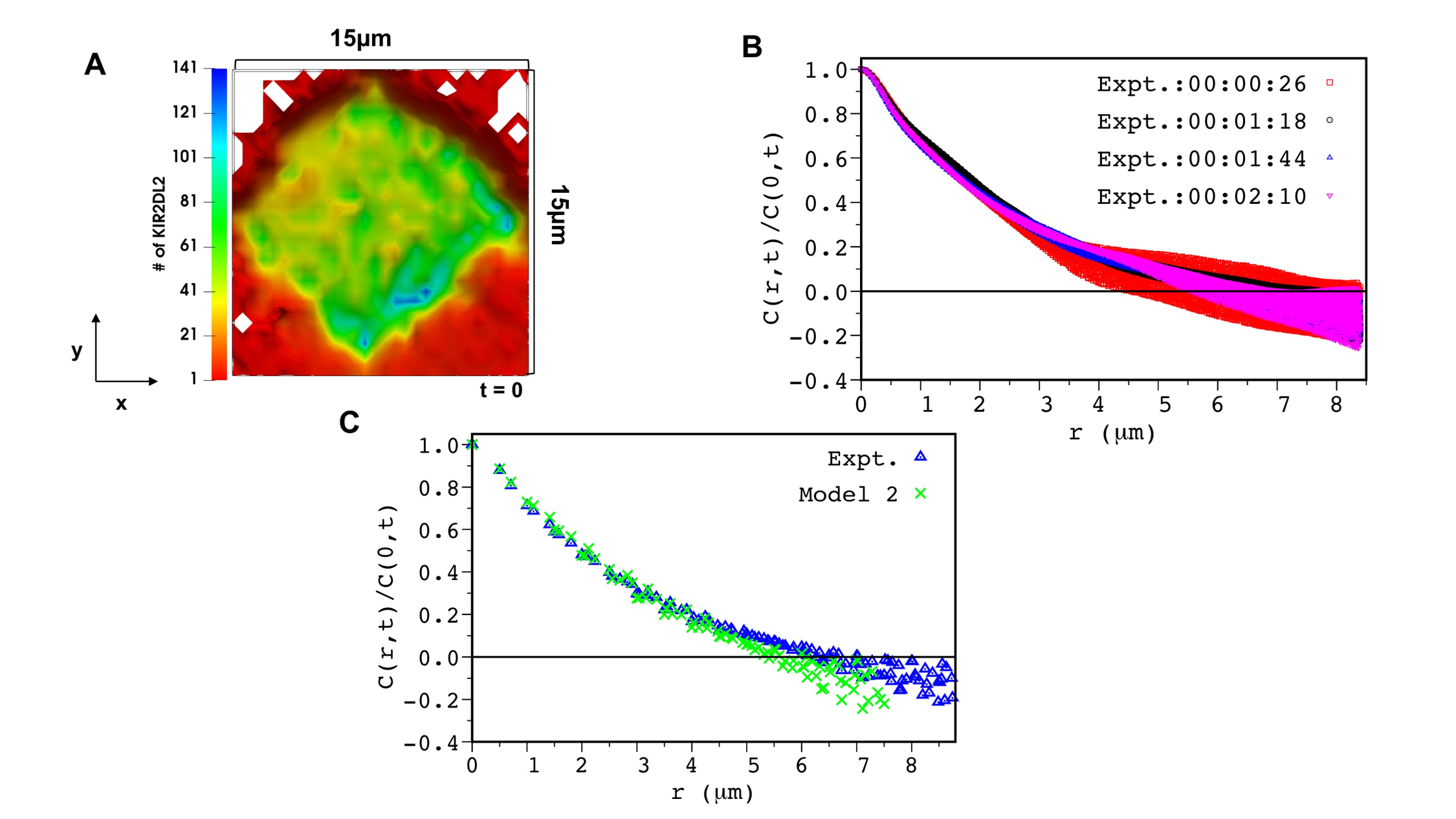

Supplement: S5 Fig — (A) Shows initial configuration of KIR2DL2 at t = 0 in our simulations based on the coarse-grained 2D image extracted for a region of interest in TIRF image (Fig 4 in Ref. [6]) of KIR2DL2-GFP at t = 1 min 44 sec to match the minimum length scale (~ 0.5 μm) of spatial resolution in our model. (B) C(r,t)/C(0,t) calculated for intensities of KIR2DL2-GFP extracted from TIRF experiments (Fig 4 in Ref. [1]) at times prior and ~30s post stimulation by HLA-C ligands (UV irradiation at t = 1min 44 seconds (00:01:44), blue filled circles) for the region of interest from TIRF images. (C) C(r,t)/C(0,t) calculated for intensities of KIR2DL2-GFP for the region of interest from TIRF image (blue empty triangles) and after coarse-graining (corresponding to image A) to obtain the minimum scale length resolution of our simulation box (green cross). (TIF) [file pcbi.1010114.s005.tif]

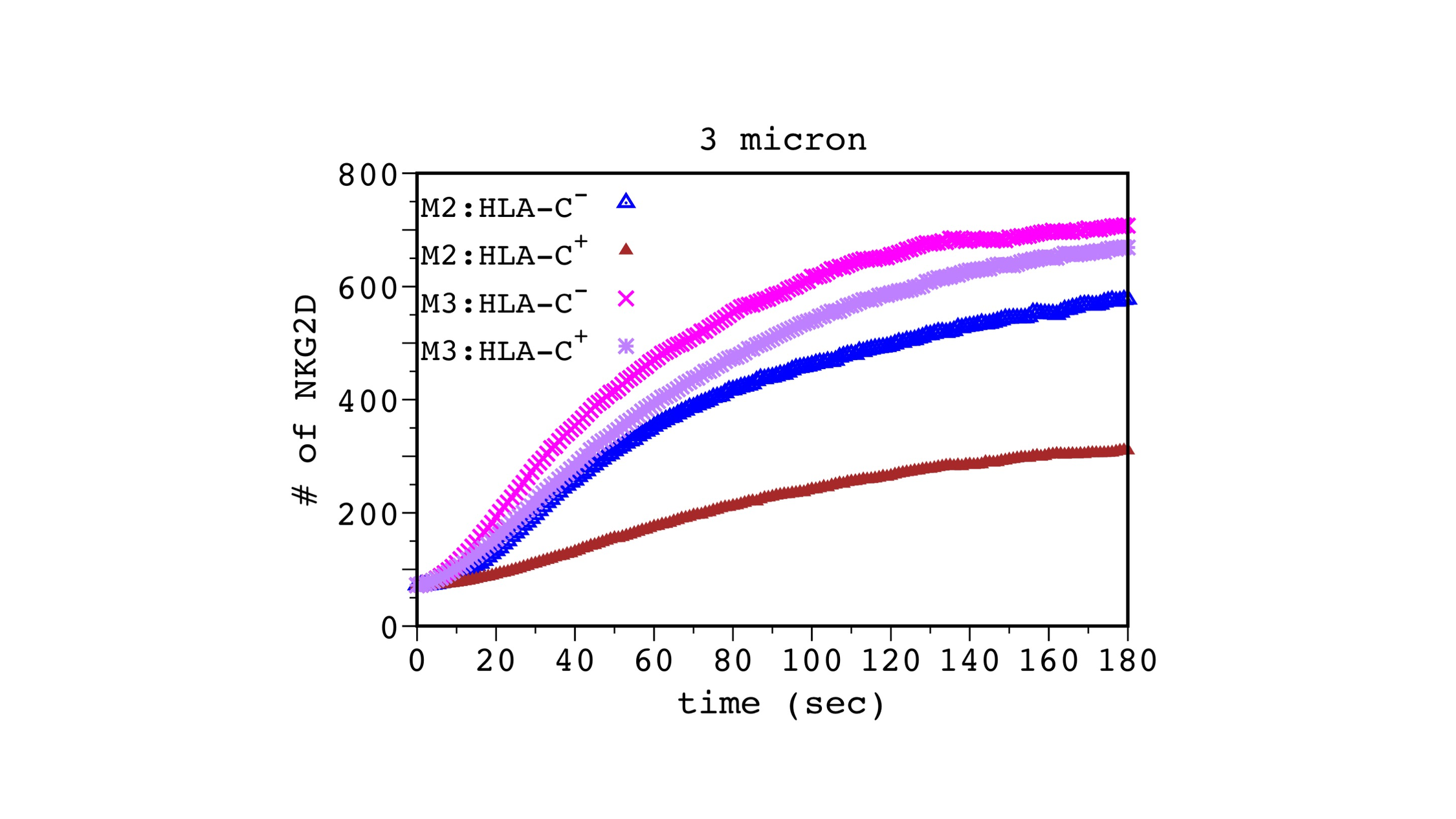

Supplement: S6 Fig — Shows the number of NKG2D molecules in a 3μm ×3μm area at the center of the simulation box for Model 2 (M2) and Model 3 (M3) in the presence (filled brown triangle for M2; purple asterisk for M3) and absence (empty blue triangle for M2; pink × for M3) of inhibitory ligands (HLA-C). The parameters for the simulation are set to the best-fit values. The NKG2D concentrations are averaged over 200 different configurations. KIR2DL2 inhibition abrogates centripetal movements of NKG2D receptor clusters for Model 2 but not for Model 3. (TIF) [file pcbi.1010114.s006.tif]

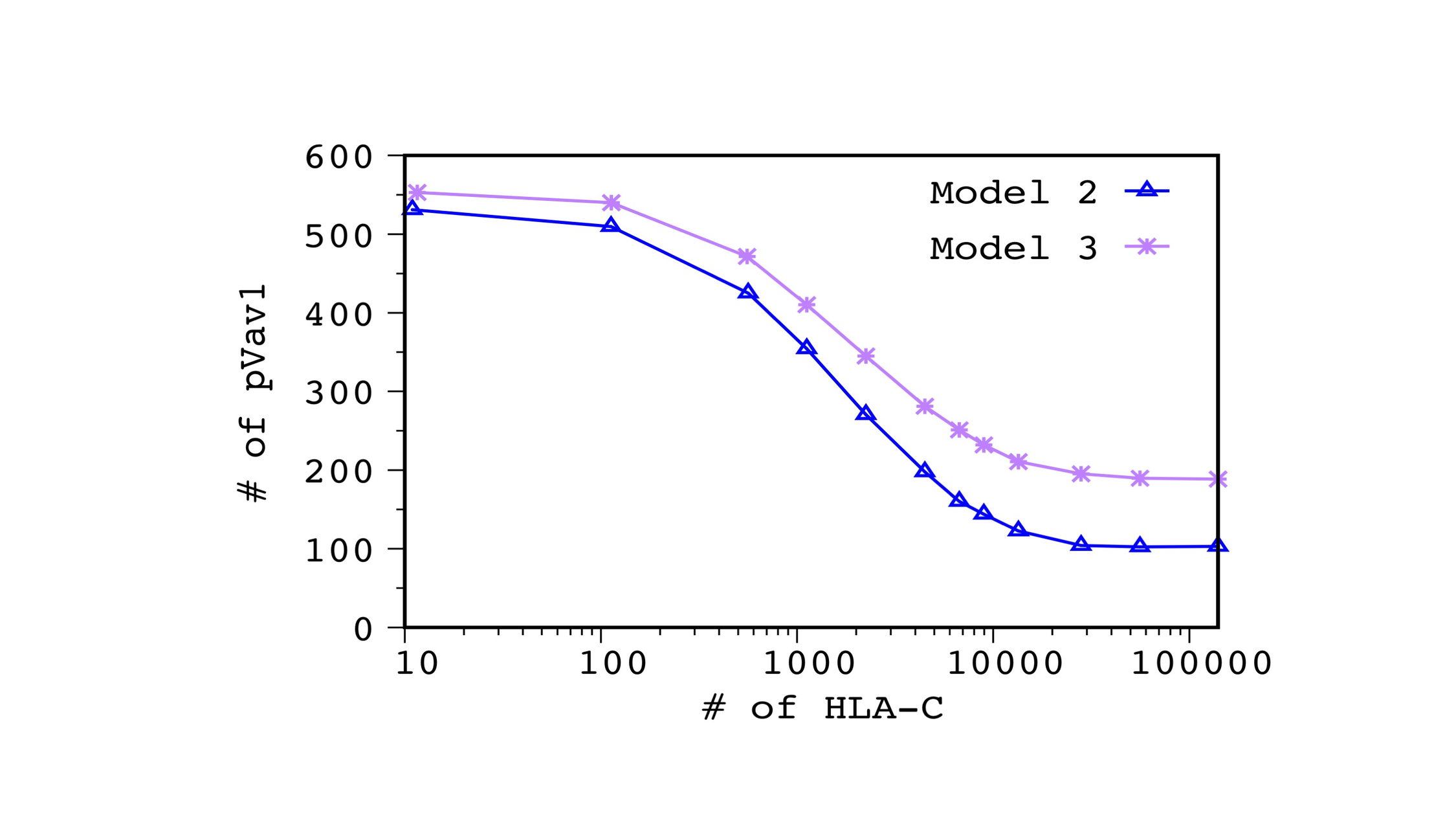

Supplement: S7 Fig — We co-localized KIR-2DL2-HLA-C complexes in a ring pattern at t = 0 in our simulations as shown in Fig 6A. NKG2D, ULBP3 and other parameters are set as described in the section pertaining to Fig 6. The figure shows variation of total number of pVav1 at t = 1 min with increasing HLA-C concentrations for Model 2 (empty blue triangle) and Model 3 (purple asterisks). The decrease in pVav1 is higher in Model 2 compared to Model 3. The pVav1 values shown were obtained by averaging over 50 configurations for each HLA-C dose. (TIF) [file pcbi.1010114.s007.tif]

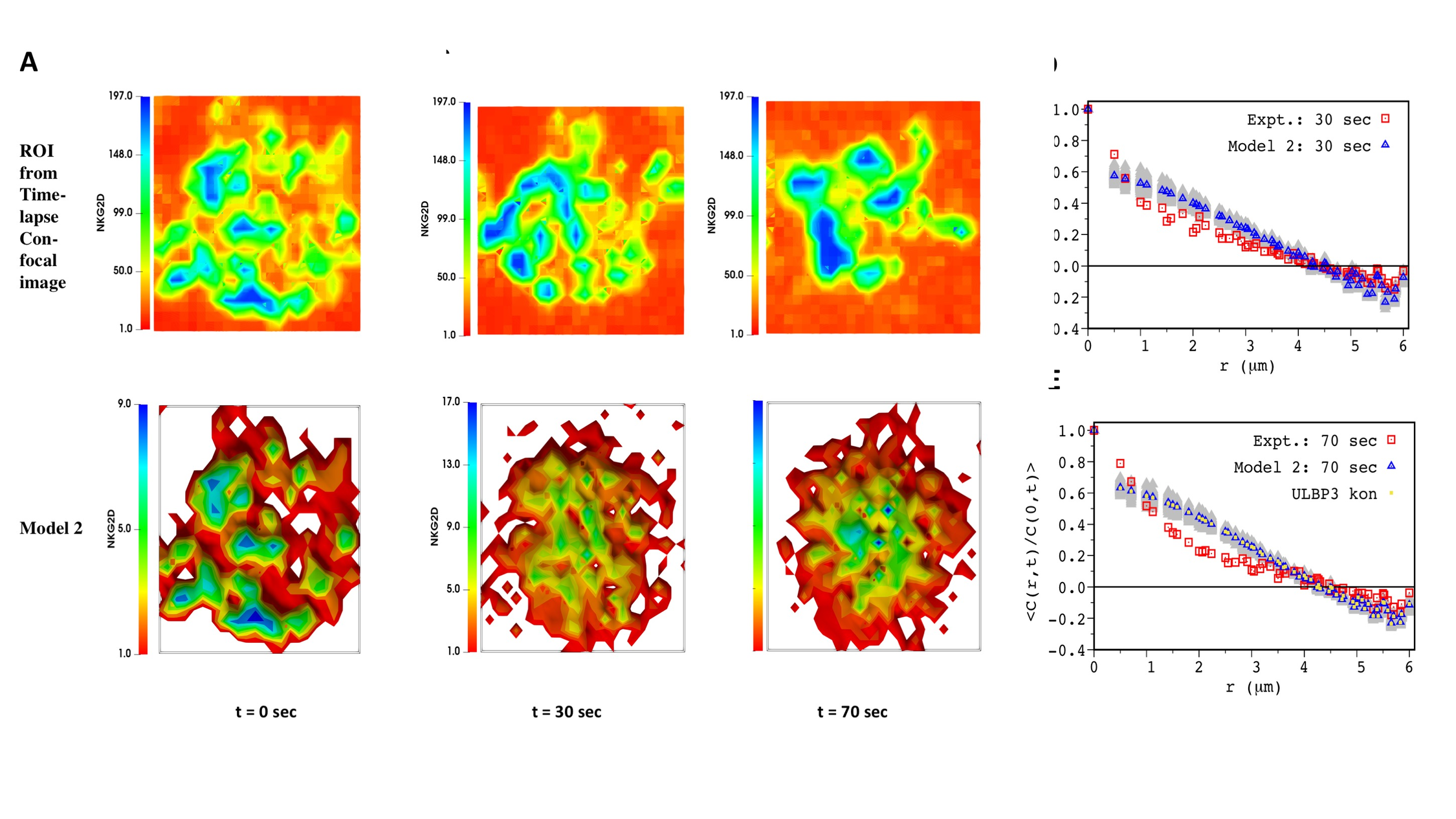

Supplement: S8 Fig — We extracted two-dimensional fluorescence intensity of NKG2D-GFP in single NKL published in Ref. [7] following an image extraction method described in the Materials and Methods section. The NKLs in Ref. [7] were stimulated by MICA ligands on target Daudi/MICA cells and NKG2D-GFP molecules were imaged using confocal microscopy. We chose a region of interest in the extracted image and coarse-grained it to match the minimum length scale (0.5 μm) of our simulation. We used the confocal data at t = 0 (A; top) to create the initial configuration of NKG2D receptors in the model (Model 2). The rest of the parameters are initialized as described in the Materials and Methods sections. We simulated the initial configuration in our model (Model 2) using the best fit parameters (Table 3) we obtained for Model 2 using the TIRF imaging data in Ref. [6]. The spatial organization of NKG2D in the simulation (top panels in (A)-(C)) and experiments (bottom panels in (A)-(C)) at t = 30s and t = 70s were compared using the two-point correlation function (D-E). The two-point correlation functions in the simulations are averaged over an ensemble of 100 configurations. The values for the correlation function for individual simulation trajectories are shown in grey. (TIF) [file pcbi.1010114.s008.tif]

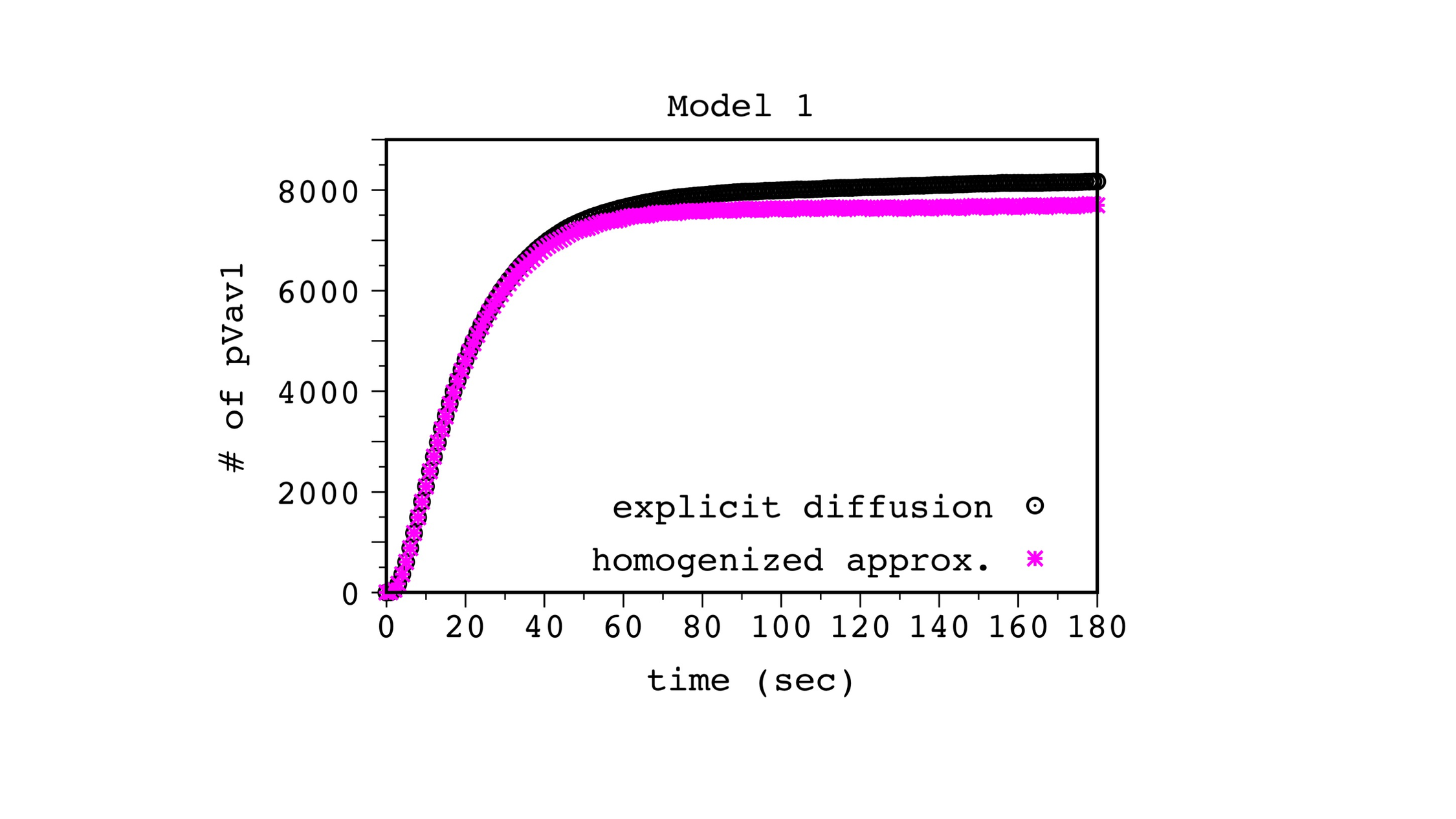

Supplement: S9 Fig — Shows the total number of pVav1 with time in Model 1 when diffusion of cytosolic molecules is introduced explicitly in the simulations (black, empty circle), which is compared against our simulations approximating explicit diffusion with homogenization of those molecules at discrete times (magenta, asterisk). The pVav1 concentrations are averaged over 200 different configurations. The parameters for the simulation are set to the best-fit values from PSO. (TIF) [file pcbi.1010114.s009.tif]

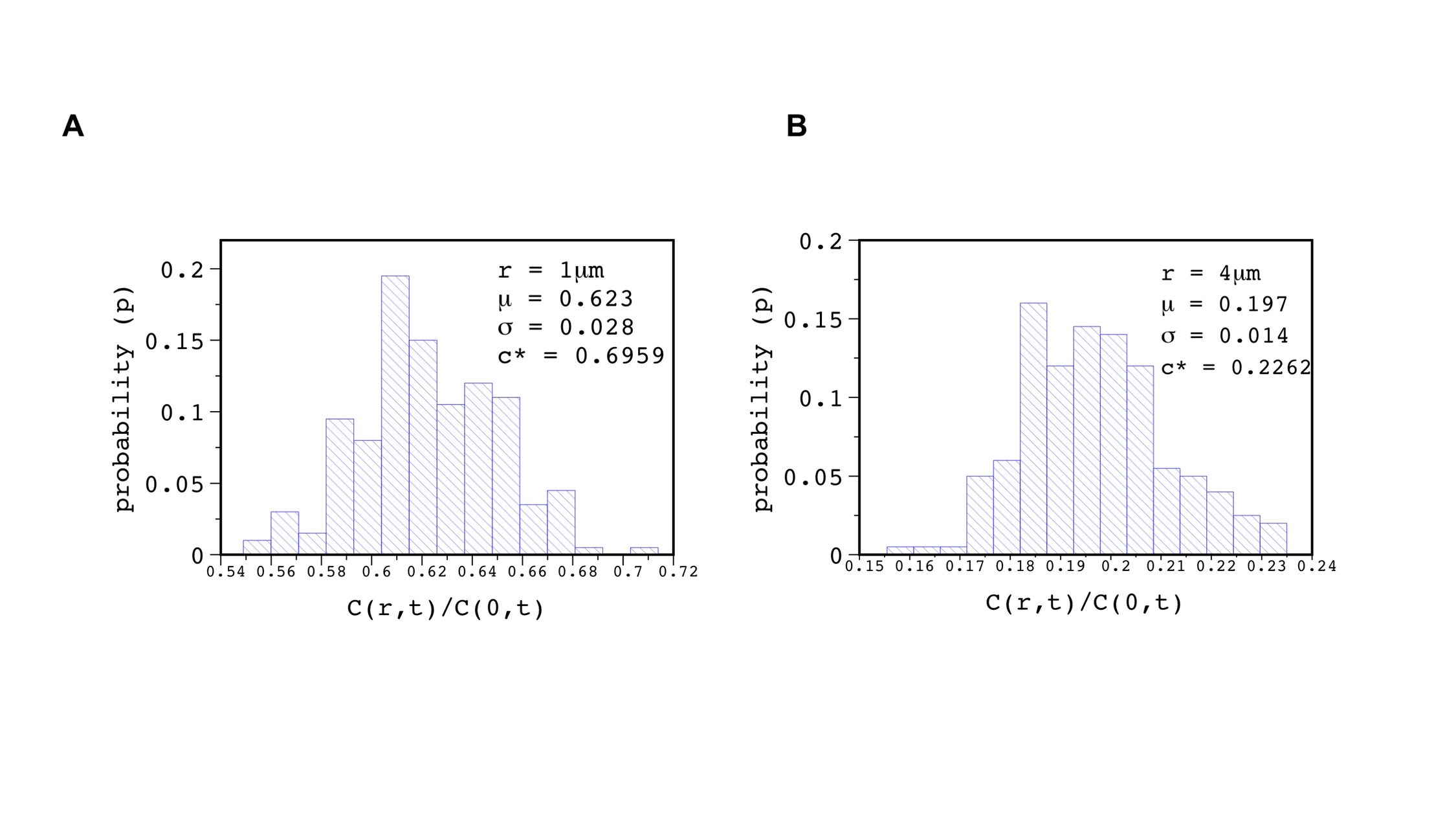

Supplement: S10 Fig — Shows probability distribution function (pdf) of C(r,t)/C(0,t) at t = 1min for (A) r = 1μm and (B) r = 4μm for Model 2. The parameters for the simulation are set at the best-fit value from our PSO. The pdf is calculated for an ensemble of 200 configurations. μ and σ represent the mean and standard deviation of the pdf, respectively. c* denotes the values of the C(r,t)/C(0,t) at t = 1min at the r values shown in (A) and (B) for the best-fit NKG2D configuration obtained in the PSO. (TIF) [file pcbi.1010114.s010.tif]

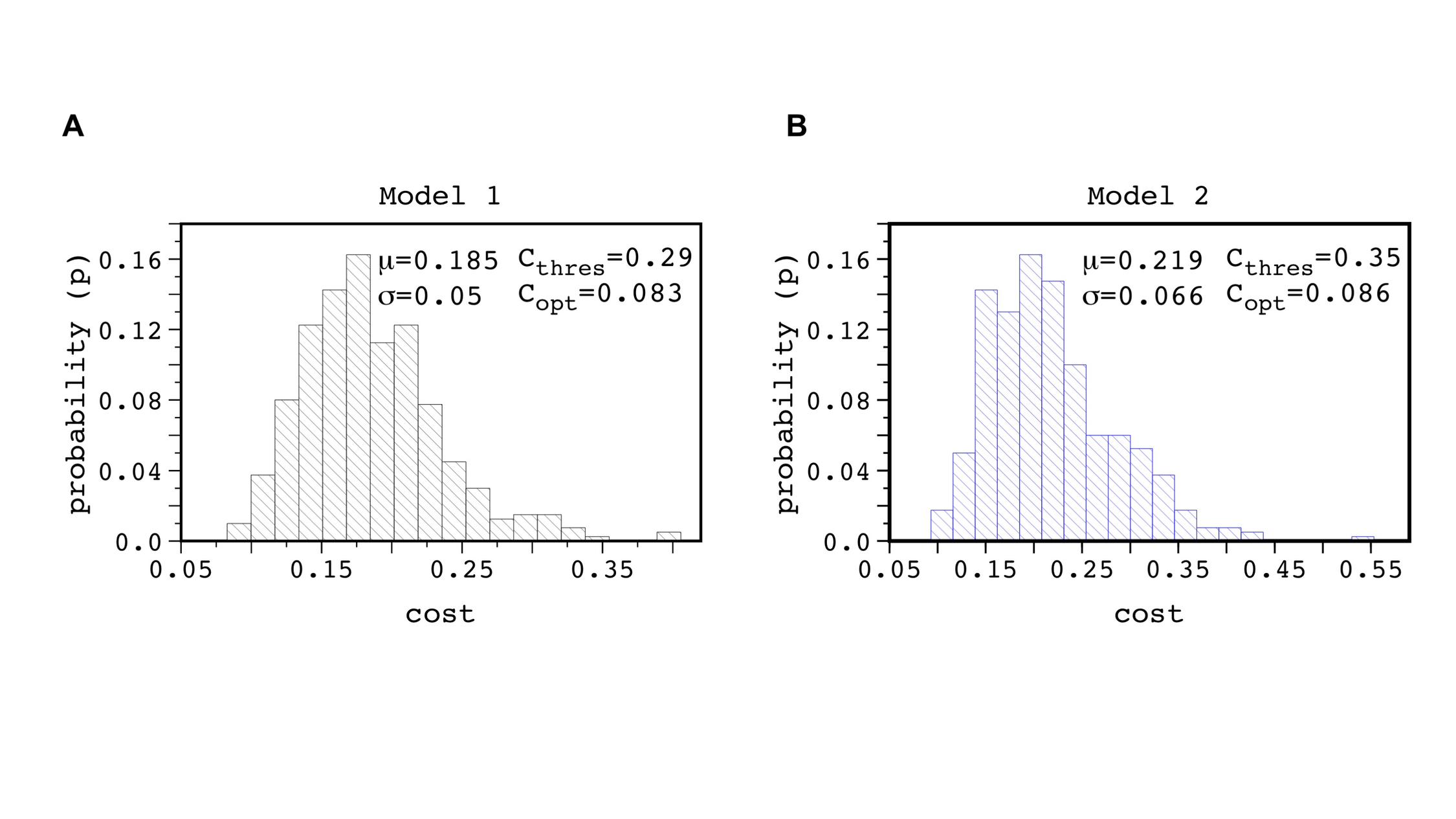

Supplement: S11 Fig — η denotes the random variables associated with intrinsic noise fluctuations. The pdf is calculated for an ensemble of 400 configurations. μ and σ denotes the mean and standard deviation for Ccost(θmin; η, n0) for the corresponding model. Copt represents the optimum (minimum) cost function Ccost(θmin; η = ηpso_otim; n0) estimated by PSO for each model. The uncertainty in our estimated parameters θmin is estimated to lie within the interval 0≤ Ccost≤ Cthres, where Cthres is obtained as Cthres = Copt +2σ. (TIF) [file pcbi.1010114.s011.tif]

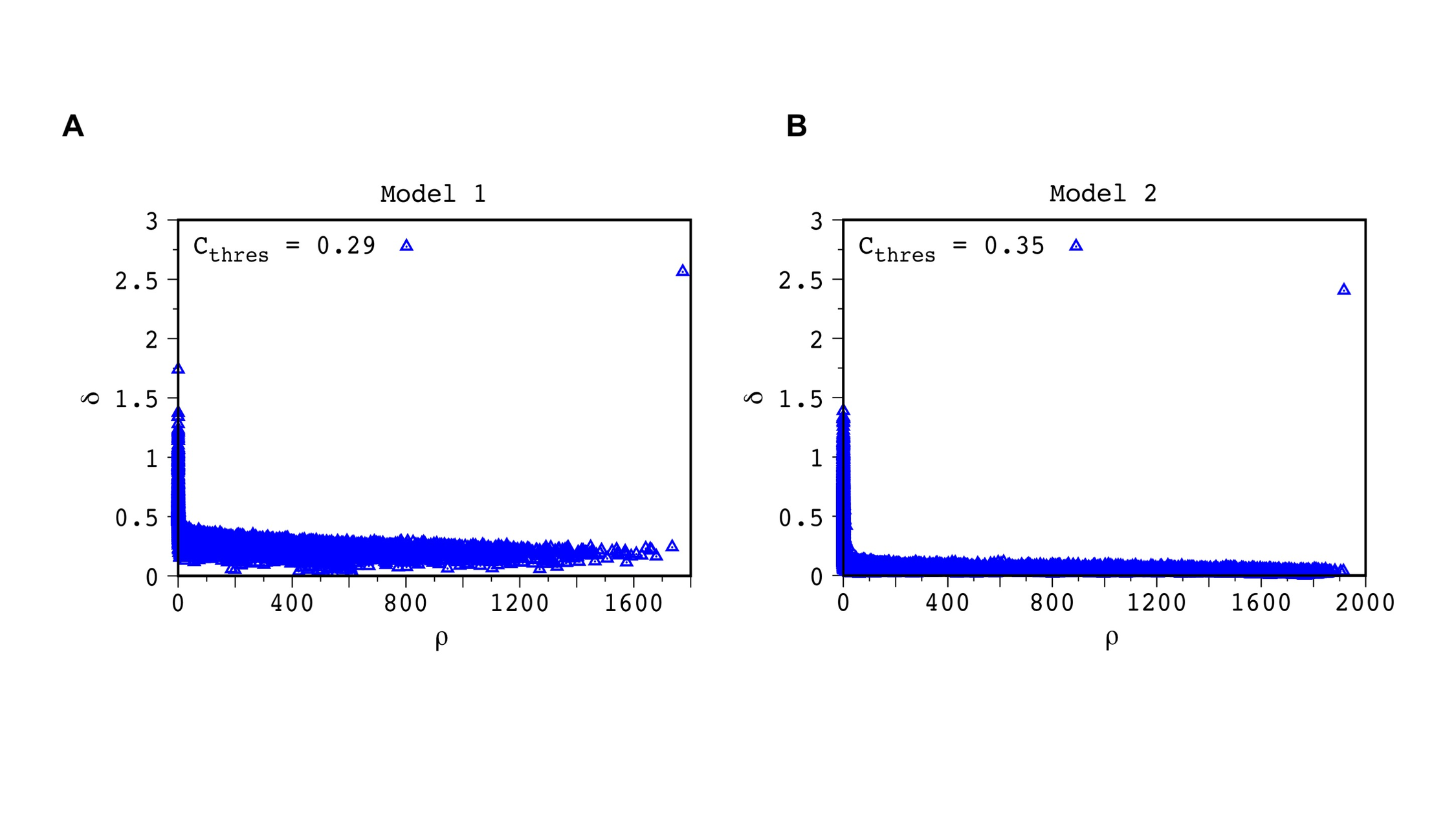

Supplement: S12 Fig — The points having relatively large local density (ρ) and high value of δ are considered to be the cluster centers defined in Ref. [92]. For both, Model 1 (A) and Model 2 (B), we observed only one such cluster center to exist implying the presence of a single minimum cost function in the parameter range explored by the PSO. The decision graph was computed for parameters θ in the solution space having cost function, Ccost such that 0≤ Ccost≤ Cthres,, where Cthres = 0.29 and 0.35 for Model 1 and Model 2, respectively. The parameter dc in the Density Peak Clustering algorithm in Ref. [92] is calculated such that the average number of neighbours is 2% of the total number of points in the data set. (TIF) [file pcbi.1010114.s012.tif]

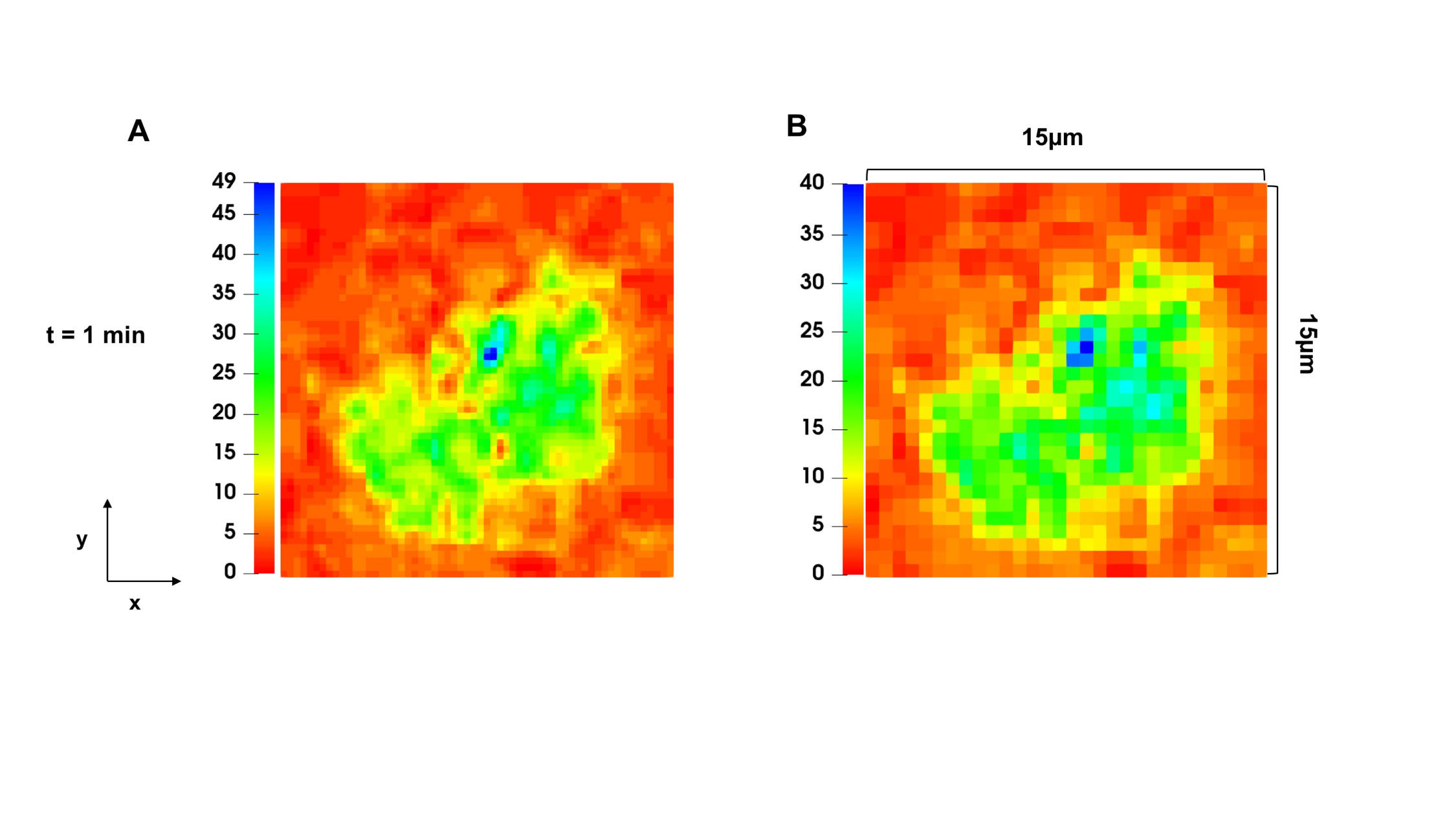

Supplement: S13 Fig — (A) Region of interest extracted from S4 Fig at t = 1min in Ref. [6]. (B) Coarse-grained image of region of interest in (A) to obtain the minimum scale length resolution (~ 0.5 μm) in our simulations. The color bar shows the extracted intensities of Dap10-mCherry from the TIRF experiments in Ref. [6]. (TIF) [file pcbi.1010114.s013.tif]

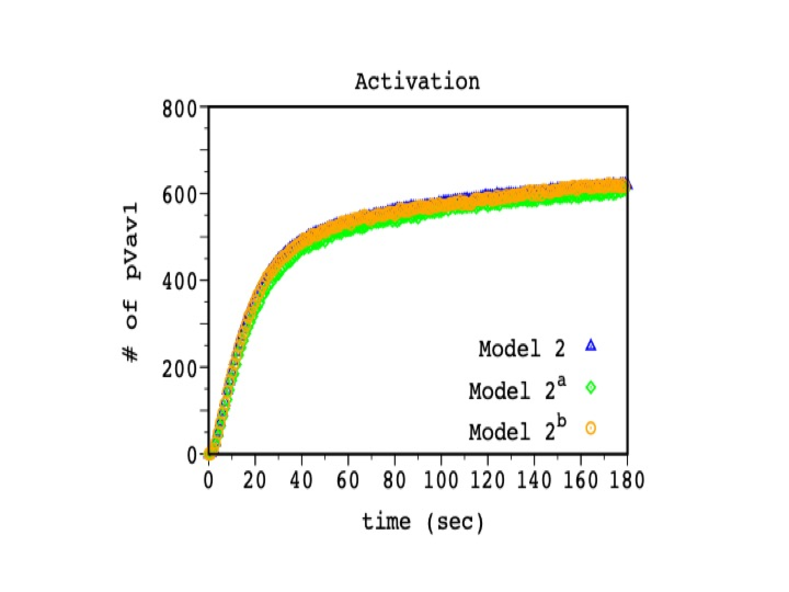

Supplement: S14 Fig — Shows the total number of pVav1 with time for Model 2 for the values of these rates estimated in our PSO (blue triangle), estimations from the literature for SFK:NKG2D (orange circle), and Lck:CD3ζ (green diamond). The estimated values are shown in S1 Text. The pVav1 concentrations are averaged over 50 different configurations and show no appreciable differences for the three sets of rates that were used. (TIF) [file pcbi.1010114.s014.tif]

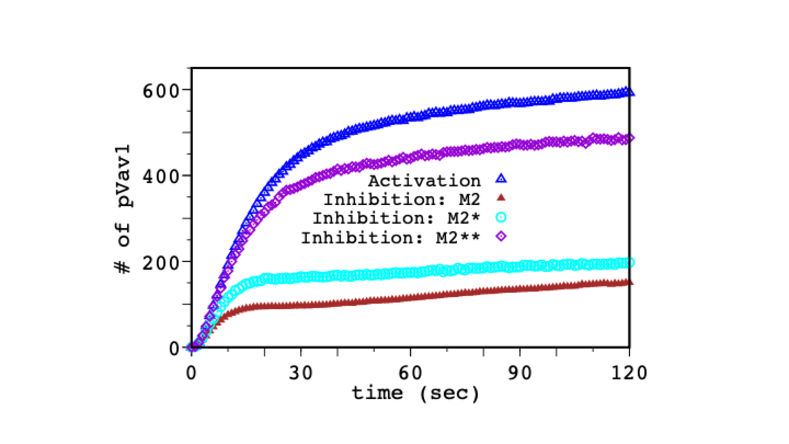

Supplement: S15 Fig — Shows pVav1 kinetics in the presence of activating ULPB3 and inhibitory HLA-C ligands. The kon value was decreased 10× (cyan circles) and 150× (purple diamonds) from the value (6.11 μM-1 s-1) used in Model 2 (brown triangle). The pVav1 kinetics in with activating ULBP3 and in the absence of inhibitory ligands is shown as a reference (blue empty triangle). KIR2DL2 is distributed in the simulation box following the data extracted from TIRF imaging at 1 min 44 sec in Fig 4 of Ref. [6]. The pVav1 concentrations are averaged over 50 different configurations. (TIF) [file pcbi.1010114.s015.tif]

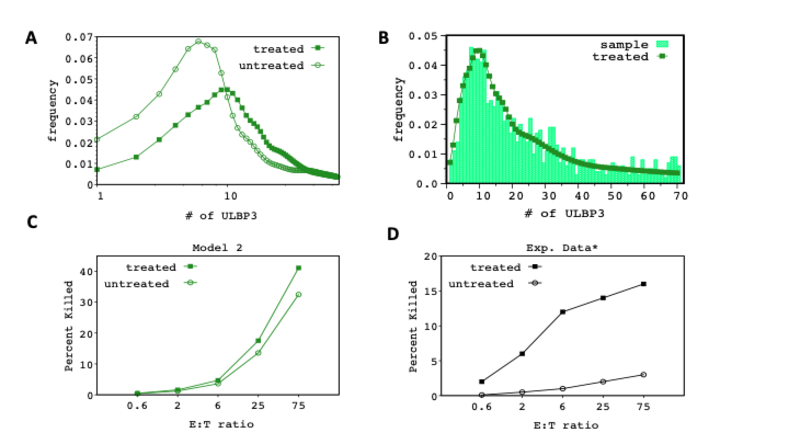

Supplement: S16 Fig — (A) Shows normalized distribution of NKG2D ligands in the treated and untreated target cells extracted from the Fig. 2d in Ref. [63] using a graphing software. (B) Distribution of NKG2D ligands when a sample of 1000 target cells (filled bars) are drawn from the normalized distribution for the case of treated target cells shown in dark-green filled squares. (C) Percentage lysis for the population of 1000 treated or untreated target cells. (D) Percentage lysis of target cells corresponding to data from Fig. 2d in Ref. [63]. (TIF) [file pcbi.1010114.s016.tif]
