# Supplementary material for: Spatially resolved in silico modeling of NKG2D signaling kinetics suggests a key role of NKG2D and Vav1 Co-clustering in generating natural killer cell activation
Source: PLoS Comput Biol. 2022 May 18;18(5):e1010114. doi: 10.1371/journal.pcbi.1010114 (PMC9154193; doi:10.1371/journal.pcbi.1010114)
Supplement: S1 Text — (DOCX) [file pcbi.1010114.s017.docx]

**Estimation of rate for binding and unbinding of catalytic domain of SFK interacting with tyrosine residues in (Dap10 –NKG2D) based on the estimated values of k_off_, K_m_, k_cat_ from literature.**

The rates $k_{\mathrm{on}\left\{ Dap10:SFK \right\}}$ and $k_{\mathrm{off}\left\{ Dap10:SFK \right\}}$were not directly available for NKG2D-DAP10 or CD3ζ, so we estimated those using the formula, k_on_= (k_off_+k_cat_)/K_m_, from the reported values of K_m_ (K_m { NKG2D:SFK_ _}_ = 2.92 µM (Ref. [1]); K_m {CD3ζ:Lck_ _}_ = 96.548 molec.µm^-2^ (Ref. [2])), k_cat_ (k_cat {NKG2D:SFK_ _}_ = 6.61 min^-1^ (Ref. [1]); k_cat {CD3ζ:Lck_ _}_ = 6 s ^-1^ (Ref. [2])) , and k­_off_ (set to k_off {Lck:CSK}_ = 0.044 s^-1^ (Ref. [3]) for all the calculations). The reported rates in Ref. [2] are given in 2D whereas our simulation is in quasi-3D, thus, we converted the 2D rates from Ref. [2] to the unit of s^-1^ that we used to simulate reactions in a small chamber in our model. The results are shown in the table below.

|  | k_on_ | k_off_ |
| --- | --- | --- |
| PSO (our model) | 1.054 µM^-1^ s^-1^  (0.7 s^-1^ in small chamber for our simulation) | 0.006 s^-1^ |
| Case-a (CD3$\zeta$ (site A1):Lck) | 0.0626 µm^2^ s^-1^  (0.25 s^-1^ in small chamber for our simulation) | 0.044 s^-1^ |
| Case-b (NKG2D:SFK) | 0.0527 µM^-1^ s^-1^  (0.035 s^-1^ in small chamber for our simulation ) | 0.044 s^-1^ |

Next, we tested the sensitivity of pVav1 kinetics on the binding/unbinding rates ($k_{\mathrm{on}\left\{ Dap10:SFK \right\}}$ and $k_{\mathrm{off}\left\{ Dap10:SFK \right\}}$) against the values we estimated in the above table. All the three sets (PSO, Case-a, Case-b) show similar pVav1 kinetics (S14 Fig).

**References**

1. Makaryan SZ, Finley SD. Enhancing network activation in natural killer cells: Predictions from in silico modeling. Integrative Biology. 2020;12(5):109-21.
2. Rohrs JA, Zheng D, Graham NA, Wang P, Finley SD. Computational model of chimeric antigen receptors explains site-specific phosphorylation kinetics. Biophysical journal.
3. Rohrs JA, Wang P, Finley SD. Predictive model of lymphocyte-specific protein tyrosine kinase (LCK) autoregulation. Cellular and molecular bioengineering. 2016;9(3):351-67.
