# Supplementary material for: Spatially resolved in silico modeling of NKG2D signaling kinetics suggests a key role of NKG2D and Vav1 Co-clustering in generating natural killer cell activation
Source: PLoS Comput Biol. 2022 May 18;18(5):e1010114. doi: 10.1371/journal.pcbi.1010114 (PMC9154193; doi:10.1371/journal.pcbi.1010114)
Supplement: S3 Text — (DOCX) [file pcbi.1010114.s019.docx]

**Modeling cytotoxic killing assay**

We use our early-time spatiotemporal NKG2D signaling model to predict percentage lysis in NK cell cytotoxic assay reported in Gasser et al. (Ref. [1]) Since our NKG2D signaling kinetic model describes production of pVav1 at early times (~3 mins), we need to set up an approximate framework to relate pVav1 abundance at early time to lysis of target cells which occur due to many signaling and physiochemical processes which are initiated in part by pVav1. We hypothesize that the rate at which an NK cell lyses a target cell is proportional to function *f*([pVav1], *x*), where, [pVav1] represents the amount of pVav1 at t=3mins in the NK cell interacting with the target cell, and *x* represents the effect of other interactions (e.g., adhesion receptor-ligand interactions or activating signals generated by non-NKG2D ligands). In the cytotoxic assay in Ref. [1] (Fig. 2d in their manuscript), a range of effector to target cell ratios (e.g., 0.6, 2, 6, 25, 75) are considered. For an E:T ratio = n>1 (e.g., 6), we can assume on average a single target interacts with *n* NK (or effector) cells. In order to simplify calculations further, we assume a single encounter between an NK cell and a target cell leads to the lysis of the target cell. Therefore, if E and T represent the NK cell and the target cell, respectively, the lysis is described by a single reaction, E+T → E + ϕ, where the reaction rate is *k*. If the assay is carried out for a time of τ (e.g., 3 hrs for Gasser et al., Ref. [1]), the probability that a target cell encounters one of the *n* NK cells in the time interval τ is given by,

P_encounter_ (τ)= 1-*exp*(-*knτ*). We assume k∝ *f*([pVav1], *x*)=α[pVav1]g(*x*). Since, any encounter between a target cell and an NK cell leads to lysis of the target cell in our model, we have %lysis= P_encounter_(τ)×100. Next, we applied this model to predict lysis of mouse T-cell blast target cells that were treated with the DNA polymerase inhibitor aphidicolin or were left untreated. Treatment with aphidicolin moderately increases the expression of NKG2D ligands in these target cells (Fig. 2d in Ref. [1]). (A) We extracted the distribution of NKG2D ligands in the treated and untreated target cells from the Fig. 2d in Ref. [1] using a graphing software and normalized the distributions. We then drew 1000 target cells using the above distributions, and for each target cell we computed the pVav1 abundance produced in a single NK cell that interacts with the target cell. Then we used that pVav1 abundance (or [pVav1]*_i_*) induced by a target cell (#*i*) to compute P_encounter (_*_i_*_)_ for the *i*^th^ target cell using, k*_i_*=α[pVav1]*_i_*g(x). The %lysis for the target cell population is then given by, %lysis= [∑_i_ P_encounter(i)_ (τ)]*100. Since, the constant α and g(x) are unknown, we currently could predict %lysis qualitatively. Also note, our NKG2D model is developed for human NK cells, whereas Gasser et al. (Ref. [1]) use mouse NK cells. NKG2D in human NK cells are associated with the adaptor DAP10, whereas, long and short forms of mouse NKG2D are associated with adaptor DAP10 and DAP10/DAP12, respectively. We assumed the lysis in Ref. [1] are induced by the NKG2D-DAP10:ligand interactions. (B) The model predicts higher %lysis for of the treated than the untreated target cells at various E:T ratios which is qualitatively similar to that shown in Fig. 2d in Ref. [1]. Since g(*x*) and α should not vary between treated and target cells within our model we set αg(*x*) to an arbitrary constant = 2. The qualitative features of the model predictions did not vary for a wide range of αg(*x*).

**References**

1. Gasser S, Orsulic S, Brown EJ, Raulet DH. The DNA damage pathway regulates innate immune system ligands of the NKG2D receptor. Nature. 2005;436(7054):1186-90.
