# Supplementary material for: Spatially resolved in silico modeling of NKG2D signaling kinetics suggests a key role of NKG2D and Vav1 Co-clustering in generating natural killer cell activation
Source: PLoS Comput Biol. 2022 May 18;18(5):e1010114. doi: 10.1371/journal.pcbi.1010114 (PMC9154193; doi:10.1371/journal.pcbi.1010114)
Supplement: S2 Text — (DOCX) [file pcbi.1010114.s018.docx]

**Rates for de-phosphorylation of pVav1 by KIR2DL2 bound SHP1**

We thank the reviewer for the comment and for the references. We did not estimate the binding-unbinding rates for KIR2DL2 bound SHP1 with the substrate pVav1 as appropriate time course of NKG2D microclusters in the presence of inhibitory KIR2DL2-HLA-C interactions were not available in Ref. [1]. Thus, we set these rates to an arbitrary but reasonable value to evaluate the effect of inhibitory signaling in affecting NKG2D clustering and pVav1 production. We estimated the catalytic rate (k_cat_ =[s^-1^]) for SHP1 dephosphorylating pVav1 using estimations from Ref. [2] and Ref. [3] in the following way. Clemens et al. (Ref. [3]) estimates the rate of dephosphorylation for PD-1 by tethered-SHP1 as ${k^{*}}_{cat\{SHP1:PD1\}}$ = 0.04 µM^-1^ s^-1^ where, ${k^{*}}_{cat}= \frac{k_{cat}}{K_{m}}$. If we use the K_m_ (= 75 μM) given in Ref. [2] for soluble substrates interaction, we estimate k_cat_ = 3 s^-1^. This value is used in our model. As a caveat, we note that PD-1 contains one ITIM and one ITSM residues whereas, KIR2DL2 contains two ITIM residues, thus binding/unbinding rates for SH2 domains of SHP-1 could be different between PD-1 and KIR2DL2.

The values of k_on_, k_off_, and k_cat_ we used in the model for dephosphorylation of pVav1 by KIR2DL2 bound SHP-1 would also suggest the K_m_ (=k_off_+k_cat_)/k_on_) for this reaction is about 150 times smaller than that of 75μM. Since SHP-1 is bound to KIR2DL2 in our model, the effective k_on_ could be larger than that of the solution because of restriction of movements of the SHP1 molecules. The larger values of k_on_ could result in smaller K_m_ values similar to that we have used in our model. To test the sensitivity of the result with decreasing k_on_, we decreased it 10× and 150 × (produces the same K_m_ as that measured in solution), and calculated the pVav1 kinetics (S15 Fig). We found that decreasing k_on_ 10× does not change the kinetics appreciably but decreasing it to 150× does produce much less inhibition. However, it is likely the K_m_ for the KIR2DL2 bound SHP-1 will be smaller than that for soluble SHP1.

**References**

1.	Abeyweera TP, Merino E, Huse M. Inhibitory signaling blocks activating receptor clustering and induces cytoskeletal retraction in natural killer cells. J Cell Biol. 2011;192(4):675-90. PubMed PMID: WOS:000288096900012.

2.	Goyette J, Salas CS, Coker-Gordon N, Bridge M, Isaacson SA, Allard J, et al. Biophysical assay for tethered signaling reactions reveals tether-controlled activity for the phosphatase SHP-1. Science advances. 2017;3(3):e1601692.

3.	Clemens L, Kutuzov M, Bayer KV, Goyette J, Allard J, Dushek O. Determination of the molecular reach of the protein tyrosine phosphatase SHP-1. Biophysical Journal. 2021;120(10):2054-66.
