# Supplementary material for: Spatially resolved in silico modeling of NKG2D signaling kinetics suggests a key role of NKG2D and Vav1 Co-clustering in generating natural killer cell activation
Source: PLoS Comput Biol. 2022 May 18;18(5):e1010114. doi: 10.1371/journal.pcbi.1010114 (PMC9154193; doi:10.1371/journal.pcbi.1010114)
Supplement: Attachment — Submitted filename: 120122_1_rebuttal_2221895_r2dsbs.docx [file pcbi.1010114.s037.docx]

**Manuscript number:** RC-2021-01036R

**Corresponding author(s):** Jayajit, Das

## 1. General Statements [optional]

We have revised the text, carried out new simulations, and added new results to address the reviewers’ comments which we believe have improved the manuscript. The new text is highlighted in grey. We thank both reviewers for the positive comments and constructive suggestions. We provide point-by-point response to the reviewer’s comments below.

## 2. Point-by-point description of the revisions

*Reviewer #1 (Evidence, reproducibility and clarity (Required)):*

*Summary:*

*In this work Grewal and Das describe the construction of an agent based model to study the biochemical processes underlying the organisation and signalling of activatory (NKG2D) and inhibitory (KIR) receptors in NK cells. They show that their model captures the slowing of NKG2D cluster migration to the central synapse, and the result that actin inhibitors decrease Vav1 phosphorylation. Their mains conclusions are that clustering of NKG2D does not significantly enhance signalling by promoting rebinding but rather co-clustering with Vav1 is important for promoting signalling. They also suggest that centripetal movements of NKG2D clusters allow efficient signal integration and inhibition by KIR receptors.*

*Major comments:*

*- A key concern is that this work suffers from overfitting. Although based on ranges determined experimentally for similar systems, nearly every parameter in the model is estimated by PSO. Since the parameters are estimated by their ability to make the model fit a single experimental dataset (Abeyweera et al (2011) (ref 6) it shouldn't be surprising that a model parameterised in this way reproduces experimental data. With such models the deep caveat becomes that the assumptions (ie that the reactions modelled are the only important reactions and have been captured correctly in the model) are never properly tested and there is very little indication of whether it has any predictive value on other datasets. The only effort there is to determine this is the ability of the model to predict time points after 1 min in the training data.*

We thank the reviewer for raising this point. We take a parsimonious approach to build our signaling model. This is a common approach used for developing detailed but manageable” signaling kinetic models where a model contains select reactions that pertain to the experimental data the model aims to describe[1, 2]. In our case, we aimed to describe NKG2D spatial kinetics and its relation to pVav1 production which is assayed many previous experiments [2-4]. Our signaling model is similar to that of Mesecke et al.[4] where some of the reactions considered were validated against experiments. In addition, there are a large number of experiments carried out over the years that validate signaling reactions we considered here. In short, we agree our model does not capture all the signaling reactions stimulated via the NKG2D-ULPB3 binding but we account for key reactions that are pertinent for describing specific experiments (e.g., NKG2D clustering, pVav1 abundance in cyto-D treatment) with our model. We have revised the text to make this point (lines 135 -144) which is also appended below.

We considered key membrane proximal biochemical signaling reactions involved in NKG2D and KIR2DL2 signaling. The specific roles of signaling proteins (adaptors, kinases, GEFs) considered here in regulating NKG2D signaling have been validated in experiments and modeling over the years. A selection of the pertaining literature is cited below and in Table 1. Similar to Mesecke et al.[4], we take a parsimonious approach in creating the NKG2D signaling network where key signaling reactions are considered. This approach is widely used for developing “detailed but manageable” signaling kinetic models where a model contains select reactions that pertain to the experimental data the model aims to describe[1]. Below we describe signaling reactions and specific approximations considered in the model.

We did not subject our model for any standard test for overfitting (e.g., 10-point cross validation) as setting specific parameter values to zero here will remove specific biological process we intended to capture in the model. For example, setting K=0 (rule #16) will make the mobility of the NKG2D microclusters pVav1 independent. However, such a model (our Model 3) would be able to describe clustering of NKG2D measured in the TIRF experiments with a different set of values for the parameters, however, this model does not capture the interplay between NKG2D signaling and the motility of NKG2D microclusters. Thus, Model3 is unable to describe abrogation of the motility of NKG2D microclusters in the presence of inhibitory ligands as observed by Abeyweera et al.[5]. To reiterate, our aim was to create a “simple but manageable” model that can capture to relevant biological processes for NKG2D spatiotemporal signaling and not to create a minimal model with potential unrealistic interactions that can describe the TIRF imaging data.

In order to test the utility of our model for predicting other datasets we used our model (Model 2) to predict clustering of NKG2D in confocal experiments [2] where NKG2D receptors in NKL cell line were stimulated by MICA ligands on Daudi target cells. Compared to the TIRF experiments this is a very different situation where NKG2D ligands are stimulated by a different ligand (MICA) presented on target cells. We find our model can predict the clustering of NKG2D reasonably well until 70s (new Figure S8; also appended below). This test increases confidence on the ability of the model to predict spatiotemporal kinetics of NKG2D signaling in other experiments.

**Figure S8. Model prediction for NKG2D spatial clustering at later times are in agreement with live cell confocal microscopy reported in Ref. [2] :** We extracted two-dimensional fluorescence intensity of NKG2D-GFP in single NKL published in Ref. [2] following an image extraction method described in the Materials and Methods section. The NKLs in Ref. [2] were stimulated by MICA ligands on target Daudi/MICA cells and NKG2D-GFP molecules were imaged using confocal microscopy. We chose a region of interest in the extracted image and coarse-grained it to match the minimum length scale (0.5 μm) of our simulation. We used the confocal data at t=0 (A; top) to create the initial configuration of NKG2D receptors in the model (Model 2). The rest of the parameters are initialized as described in the Materials and Methods sections. We simulated the initial configuration in our model (Model 2) using the best fit parameters (Table 3) we obtained for Model 2 using the TIRF imaging data in Ref. [5]. The spatial organization of NKG2D in the simulation (top panels in (A)-(C)) and experiments (bottom panels in (A)-(C)) at t=30s and t=70s were compared using the two-point correlation function (D-E). The two-point correlation functions in the simulations are averaged over an ensemble of 100 configurations. The values for the correlation function for individual simulation trajectories are shown in grey.

*- It seems that the interpretation is that in the model pVav1 levels are considered "signalling" in general. Obviously pVav1 is just one of the important molecules in the cascade of proximal signalling reactions that occur downstream of ITAM phosphorylation on activating receptors. There needs to be much more nuance given to the interpretation of the model outputs.*

Vav1 plays a key role in proximal NK cell signalling. 1) Phosphorylation of Vav1 leads to F-actin reorganization which regulates NKG2D microcluster movements, thus, forming a link between early NK cell signalling and NKG2D spatial movements. 2) Vav1 is signalling protein where integration between activating and inhibitory signals occurs, i.e., Vav1 is phosphorylated by Syk kinases recruited by NKG2D-DAP10 and dephosphorylated by SHP-1 bound to inhibitory KIR2DL1 receptors. Therefore, we considered Vav1 in our model. Another motivation to include Vav1 was to compare our results with experiments by Endt et al. The authors reported a decrease in pVav1 when actin polymerization was inhibited by cytochalasin D. We agree with the reviewer that there could be other proteins along with Vav1 which play important roles in downstream signalling (e.g., PI3K), however, as we responded above, our aim was to create a detailed but manageable signalling model that describes key signalling reactions and can be compared with available experiments, thus we do not include those additional proteins. However, given a question of interest, our model can be extended easily to describe additional proteins. We have added text to address the comment.

We represented multiple SFKs (e.g., Lck, Fyn, Lyn, Yes) in NK cells for simplifying models. There are differences in substrate specificities[6] for SFKs in T cells. It will be straightforward to extend our models to include multiple SFKs if similar differences are found in NK cells. Similarly, proximal signaling proteins such as PI3K[7] were not included in the model to keep the model simple and focused on the questions of interest addressed here.

*- It is stated that a strength of the study is the estimation of parameters that might otherwise be difficult to measure. This should come with a very large caution that these parameters are only useful and accurate if the assumptions of the model are valid and accurate, and as pointed out above this is not rigorously tested. A reader might only look at the fact that several of the parameters vary to a large extent between the 3 models tested, and thus depend strongly on the unlying assumptions of each model, to see that they may only have validity within the model system.*

The signalling models considered here represent approximations of the signalling network in NK cells. The models contain a small number of key reactions and provide an effective or coarse-grained description of the signalling kinetics. The signalling reactions considered have been validated in previous models ([4]) as well as in many experiments. In addition, it is expected that two models that contain different inter-linked processes but fit the same kinetic data will give rise to different model parameter values. Our goal was to compare the roles of ligand rebinding and Vav1-NKG2D co-clustering in regulating proximal NK cell signalling. Since the roles of the above mechanisms depend on the model parameters we wanted to compare those in parameter regimes where the models fit the experimental data. We tested the applicability of our parameter values for describing other experimental data in the revised manuscript where we have shown that our model (Model 2) can capture NKG2D clustering in NKLs stimulated by a different ligand (MICA) presented on target cells (Daudi cells) (new Figure S8). This is a very different experiments compared to the TIRF experiments in Abeyweera et al.[5] which provides confidence on the applicability of our parameter estimation for describing other systems.

*- It is not clear what biology the different models are meant to be investigating. In particular, in Model 2 & 3 Case II the HLA-C is presented in a ring pattern, which seems odd. Under what biological context would the HLA-C be presented to NK cells in a ring pattern? In the quoted Almeida and Davis (2006) (ref 34) paper the authors describe 4 different patterns of HLA-C within the NK-target cell immunological synapse - ring structures were one of the least abundant of these structures. Firstly, the authors also observed these patterns after incubating cells together for 10 mins, which is much later than the timepoints modelled in this work. Secondly, and more importantly, these structures formed as a consequence of KIR ligation, rather than the cell presenting HLA-C in a ring to the NK cell as the authors seem to suggest.*

We thank the reviewer for the comment. We have revised the text and carried out a new simulation to address this point. We append the text below.

lines 370 - 374

The ring pattern could represent HLA molecules on engineered surfaces or on target cells. Almeida et al. [8] found HLA-C molecules to be organized in a ring pattern on target cells after NK cells are incubated with target cells for 10mins. The pattern formation in part is initiated by binding of inhibitory KIR2DL2 and HLA-C, and KIR2DL2 co-localizes with HLA-C in such clusters.

lines 390-392

We also carried out the above comparison when KIR2DL2 molecules are co-localized with HLA-C in the ring pattern to represent HLA-C clustering in target cells interacting with NK cells [8]. The results (Figure S7) were similar to that in Figure 6C.

Appended Figure S7.

**Figure S7. Effect of HLA-C spatial patterning on target cells on signal integration in NK cells:** We co-localized KIR-2DL2-HLA-C complexes in a ring pattern at t=0 in our simulations as shown in Figure 6A. NKG2D, ULBP3 and other parameters are set as described in the section pertaining to Figure 6. The figure shows variation of total number of pVav1 at t =1 min with increasing HLA-C concentrations for Model 2 (empty blue triangle) and Model 3 (purple asterisks). The decrease in pVav1 is higher in Model 2 compared to Model 3. The pVav1 values shown were obtained by averaging over 50 configurations for each HLA-C dose.

*Minor comments:*

*- Numbering the lines would make referring to specific places in the text much easier*

We have added line numbers.

*- Many sentences are too long and convoluted, requiring at least two reads to be comprehensible. For example the first sentence of the second paragraph in the "Model Development" section.*

Thank you for pointing this out. We have revised the manuscript throughout to address the comment.

*- In Table 1 the reactions in column 2 and the estimates in column 3 do not line up, making it difficult to easily determine which is referring to which.*

We appreciate the comment by the reviewer. The text has been modified accordingly.

*Reviewer #2 (Evidence, reproducibility and clarity (Required)):*

*The paper by Das and colleagues presents in silico-generted data for NK cell synapse formation and the role of microclusters involving NKG2D. How NK cell signalling is regulated is a key question in NK cell biology, not the least because of the key role of inhibitory receptors in this process. The paper makes a convincing case based on hypothesis-driven modeling that microsluster between NKG2D and Vav-1 plays a more important role than continuous rebinding of NKG2D ligands in the creation of phosphorylated for of Vav-1. The paper is clearly written and the rational underlying the modeling is pedagogically set and understandable for readers not experts in modeling. The data is firmly anchored in previous experimental evidence.*

We appreciate the positive comments.

*The one comment I have is to which extent the inclusion of only one tyrosine kinase (called SFK) simplifies the model too much, given that in NK cell signaling, multiple kinases are thought to operate sequentially and also in parallel. Perhaps this is not a limitation of the model, but the unavoidable simplifications that are needed could be elaborated on more.*

Thank you for pointing this out. We have revised the text at several places to address this.

Lines 149 -150

NK cells contain several Src family kinases including Lck, Fyn, Lyn, and Yes[9].

…

lines 154 - 155

a kinase molecule (SFK) represents multiple Src family kinases

lines 480 - 485

We represented multiple SFKs (e.g., Lck, Fyn, Lyn, Yes) in NK cells for simplifying models. There are differences in substrate specificities[6] for SFKs in T cells. It will be straightforward to extend our models to include multiple SFKs if similar differences are found in NK cells.

*Reviewer #2 (Significance (Required)):*

*The results provide fuel for further experimentation to understand NK cell synapse biology and NK cell signaling. The paper is suitable for immunologists, whoc will be able to understand what has been done, and also for modelers of course.*

We appreciate the positive comment.

1.	Goldstein B, Faeder JR, Hlavacek WS. Mathematical and computational models of immune-receptor signalling. Nature reviews Immunology. 2004;4(6):445-56. Epub 2004/06/03. doi: 10.1038/nri1374

nri1374 [pii]. PubMed PMID: 15173833.

2.	Brown ACN OS, Dobbie IM, Alakoskela JM, Parton RM, Eissmann P, et al. . Remodelling of Cortical Actin Where Lytic Granules Dock at Natural Killer Cell Immune Synapses Revealed by Super-Resolution Microscopy. . PLoS biology. 2011;9(9). doi: doi: ARTN e1001152

10.1371/journal.pbio.1001152.

3.	Endt J MF, Almeida CR, Urlaub D, Leung R, Pende D, et al. . Inhibitory receptor signals suppress ligation-induced recruitment of NKG2D to GM1-Rich membrane domains at the human NK cell immune synapse. . Journal of Immunology. 2007;178(9):5606-11.

4.	Mesecke S UD, Busch H, Eils R, Watzl C. . Integration of activating and inhibitory receptor signaling by regulated phosphorylation of Vav1 in immune cells. . Sci Signal. 2011;4(175):ra36. Epub Epub 2011/06/03. doi: doi: 10.1126/scisignal.2001325.

5.	Abeyweera TP ME, Huse M. . Inhibitory signaling blocks activating receptor clustering and induces cytoskeletal retraction in natural killer cells. . J Cell Biol 2011;192(4):675-90.

6.	Shah NH, Löbel M, Weiss A, Kuriyan J. Fine-tuning of substrate preferences of the Src-family kinase Lck revealed through a high-throughput specificity screen. Elife. 2018;7:e35190.

7.	Segovis CM, Schoon RA, Dick CJ, Nacusi LP, Leibson PJ, Billadeau DD. PI3K links NKG2D signaling to a CrkL pathway involved in natural killer cell adhesion, polarity, and granule secretion. The Journal of Immunology. 2009;182(11):6933-42.

8.	Almeida CR, Davis DM. Segregation of HLA-C from ICAM-1 at NK cell immune synapses is controlled by its cell surface density. The Journal of Immunology. 2006;177(10):6904-10.

9.	Colucci F, Di Santo JP, Leibson PJ. Natural killer cell activation in mice and men: different triggers for similar weapons? Nature immunology. 2002;3(9):807-13.
