# Supplementary material for: Spatially resolved in silico modeling of NKG2D signaling kinetics suggests a key role of NKG2D and Vav1 Co-clustering in generating natural killer cell activation
Source: PLoS Comput Biol. 2022 May 18;18(5):e1010114. doi: 10.1371/journal.pcbi.1010114 (PMC9154193; doi:10.1371/journal.pcbi.1010114)
Supplement: Attachment — Submitted filename: response_to_reviewers.docx [file pcbi.1010114.s038.docx]

Dear Editor,

Thank you for giving us the opportunity to submit a revised manuscript. We also thank the reviewers for the comments. We have revised the main text, carried out new simulations, and added new figures in the supplementary material to address the reviewer’s comments, which we believe improved the quality of the manuscript. Our point-by-point response to the reviewer’s comments are appended below. We hope you will find the revised manuscript suitable for publication in PLOS Computational Biology. Please do not hesitate to contact me if you need anything else.

Sincerely,

Jayajit Das

Associate Professor
Battelle Center for Mathematical Medicine
The Research Institute at Nationwide Children's Hospital
Biophysics Graduate Program and Department of Pediatrics
The Ohio State University, Columbus, OH 43205

email: das.70 @ [osu.edu](http://osu.edu); jayajit.das @ [nationwidechildrens.org](http://nationwidechildrens.org)
web:http://www.nationwidechildrens.org/jayajit-das
Phone: 614-355-5632 (work)
Fax: 614-355-2728

Response to Reviewers comments

-----------------------------------------

*Reviewer #1: This manuscript describes a spatial agent-based model for investigating the interaction between signalling and spatial organisation of receptors and adaptor proteins in the immunological synapses of NK cells. This is an important question for understanding the function of NK cells, as well as T cells that also form immunological synapses and integrate information between activating and inhibitory receptors. The authors show that their model captures key features of previous experimental imaging data of NKG2D clustering and dynamics in NK cell synapses and that co-clustering between Vav1 and NKG2D is more important for robust signalling than rebinding of NKG2D to its ligand. In their modelling Grewal and Das show that NKG2D translocation to the centre of the synapse could initiate a positive feedback of Vav1 phoshorylation, if translocation is movement is dependent on pVav1, and that this allows for efficient inhibition by KIR2DL1.*

We thank the Reviewer for the positive remarks.

*Overall the work presents an interesting hypothesis that is supported by previous microscopy studies. I have some questions surrounding parameter estimation, and suggest some extra discussion of limitations, which are outlined below:*

We thank the Reviewer for the positive comment, and we have edited the main text and carried out additional simulations to address the questions. The details are provided below.

*Suggest softening the claim in the title from "…Reveals role…" to "…Suggests role…" or "…is consistent with a role…". The authors show their model is consistent with previous work, but to establish that the mechanism they propose exists in the biology, some experimental validation of novel predictions made by the model would be required.*

We agree with the Reviewer and we have changed the title to from “.. Key role..” to “..Suggests a Key role..”.

*Table 1, Rule 2: Are these binding rates the estimate for the catalytic domain of the SFK interacting with the unphosphorylated DAP10 substrate (i.e. their ratio would be the Michealis-Menton constant KM)? If so why are the kinetics of SH2 domain for pY used to estimate this? They are different domains and completely different interactions. Others have estimated these values (*[*doi.org/10.1007/s12195-016-0438-7*](https://urldefense.com/v3/__http:/doi.org/10.1007/s12195-016-0438-7__;!!ODYTt7Uqo6LDP14!1M2dhxKOeZGCseMfKP_STmK8y79oPv8W8RGvQdMgqWKO4umRaj0H9GcCrJLvtqtZniYPZwBNiE2f$)*).*

We thank the Reviewer for pointing this out. We agree with the reviewer and have carried out calculations to check the effect of changing the binding/unbinding (k_on_/k_off_) and catalytic rates (k_cat_) catalysis of DAP10 substrates by the catalytic domain of SFK using estimated rates available in the literature, including the one suggested by the Reviewer. We find changing our estimated rates to the rates estimated or measured in the literature for catalytic domains of kinases such as Csk (substrate: tyrosine in Lck) or Lck (substrate: DAP10-NKG2D or CD3ζ) did not change the Vav1 phosphorylation kinetics appreciably (see S14 Fig). The koff rates estimated in those measurements/estimations fall within the range of PSO considered in our estimation. The kon rate estimated for the catalytic domain of Lck for binding with CD3ζ (Ref. 38 in our manuscript) is 3× smaller than that estimated in our PSO. Our PSO estimation coincides with the lower bound of the range we used, which indicates that this rate cannot be well estimated in our PSO. This is further evidenced by the large uncertainties (Table 3) we found for these parameters as well as by the results shown in S14 Fig. We have changed Table 1, added text (Supplementary Text I) and a figure (S14 Fig) in the supplementary section to address the above point.

*Table 1, Rule 13: Why is ITIM-bound SHP1:Vav1 binding kon set to 10x the Vav1:pDAP10 kon rate and set to have the same koff? Why was this parameter not estimated by PSO, given it depends on local concentrations and steric availability of substrates and enzymes tethered to receptors and is thus very difficult to measure and even estimate rationally (see DOI: 10.1126/sciadv.1601692 and DOI: 10.1016/j.bpj.2019.08.023).*

We thank the Reviewer for the comment and for the references. We did not estimate binding-unbinding rates for KIR2DL2 bound SHP1 with the substrate pVav1 as appropriate time course of NKG2D microclusters in the presence of inhibitory KIR2DL2-HLA-C interactions were not available in Abeyweera et al (Ref. [6] in our manuscript). Thus, we set these rates to an arbitrary but reasonable value to evaluate the effect of inhibitory signaling in affecting NKG2D clustering and pVav1 production. We estimated the catalytic rate (k_cat_ =[s^-1^]) for SHP1 dephosphorylating pVav1 using estimations from Clemens et al. (Ref. [47] in our manuscript) and Goyette et al. (Ref. [46] in our manuscript) in the following way. Clemens et al. (Ref. [47] in our manuscript) estimates the rate of dephosphorylation for PD-1 by tethered-SHP1 as ${k^{*}}_{cat\{SHP1:PD1\}}$ = 0.04 µM^-1^ s^-1^ where, ${k^{*}}_{cat}= \frac{k_{cat}}{K_{m}}$. If we use the K_m_ (= 75μM) given in Goyette et al. (Ref. [46] in our manuscript) for soluble substrates interaction, we estimate k_cat_=3 s^-1^. This value is used in our model. As a caveat, we note that PD-1 contains one ITIM and one ITSM residues whereas, KIR2DL2 contains two ITIM residues, thus binding/unbinding rates for SH2 domains of SHP-1 could be different between PD-1 and KIR2DL2.

The values of k_on_, k_off_, and k_cat_ we used in the model for dephosphorylation of pVav1 by KIR2DL2 bound SHP-1 would also suggest the K_m_ (=k_off_+k_cat_)/k_on_) for this reaction is about 150 times smaller than that of 75μM. Since SHP-1 is bound to KIR2DL2 in our model, the effective k_on_ could be larger than that of the solution because of restriction of movements of the SHP1 molecules. The larger values of k_on_ could result in smaller K_m_ values similar to that we have used in our model. To test the sensitivity of the result with decreasing k_on_, we decreased it 10× and 150 × (produces the same K_m_ as that measured in solution), and calculated the pVav1 kinetics (S15 Fig). We found that decreasing k_on_ 10× does not change the kinetics appreciably but decreasing it to 150× does produce much less inhibition. However, it is likely the K_m_ for the KIR2DL2 bound SHP-1 will be smaller than that for soluble SHP1.

The above response is included in Supplementary Text II and S15 Fig.

*Table 1, Rule 14: Catalytic rate on pNPP is a very poor indicator of catalytic rates on specific pY targets, which can have large variability depending on flanking sequences. For measurements of SHP1 catalytic rates on phosphotyrosine substrate see* [*doi.org/10.1016/j.bpj.2021.03.019*](https://urldefense.com/v3/__http:/doi.org/10.1016/j.bpj.2021.03.019__;!!ODYTt7Uqo6LDP14!1M2dhxKOeZGCseMfKP_STmK8y79oPv8W8RGvQdMgqWKO4umRaj0H9GcCrJLvtqtZniYPZ_6muasP$) *and others.*

We thank the Reviewer for the comment. Please check our response to the previous comment.

*Around line 489, a little more discussion about the limitation of not modelling cellular spreading and retraction, both of which are related to activating and inhibitory receptor signalling (eg in* [*doi.org/10.1083/jcb.201009135*](https://urldefense.com/v3/__http:/doi.org/10.1083/jcb.201009135__;!!ODYTt7Uqo6LDP14!1M2dhxKOeZGCseMfKP_STmK8y79oPv8W8RGvQdMgqWKO4umRaj0H9GcCrJLvtqtZniYPZ6x2JRfD$)*), and related to Vav1 activity. It would be useful for placing this work in this context. Splitting the limitations section into paragraphs would also make it more readable.*

We thank the Reviewer for the comment. We have edited the limitation of modeling section to address the above comments.

*Reviewer #2: In this paper, Grewal and Das create an agent based model of NKG2D and it’s signaling kinetics following binding with cognate ligands. As NK cells are activated and inactivated by a diverse set of signaling receptors, this work provides in silico insight into these signaling transduction pathways. Further, they add value to the field by tying capturing spatial signaling dynamics into their models.*

We thank the Reviewer for the positive comment.

*There were some concerns:
1) I would have liked to see more validation on their model on other signaling pathways, especially their modeling of biochemical signaling reactions. For example, how well would it predict findings from this paper on NKG2D:* [*doi.org/10.1038/nature03884*](https://urldefense.com/v3/__http:/doi.org/10.1038/nature03884__;!!ODYTt7Uqo6LDP14!1M2dhxKOeZGCseMfKP_STmK8y79oPv8W8RGvQdMgqWKO4umRaj0H9GcCrJLvtqtZniYPZ7HFXHXa$)*.*

This is an interesting and insightful comment. The above manuscript studied the increase in target cell lysis by mouse NK cells when abundances of NKG2D ligands are increased in target cells with DNA damage (Fig 2d in the Gasser et al. Ref. [62] in our manuscript). Since our model studies spatiotemporal early time (~3min) signaling in human cell line NKLs, exploring the ability of this model to describe cell lysis by NK cells would be a challenging but interesting direction. We attempted this within a reduced modeling framework, and our model is able to qualitatively describe the differences in lysis of target cells treated with aphidicolin or left untreated (Supplementary Text III and S16 Fig). We find this quite exciting which could point us to directions that can be used to integrate our early time single cell mechanistic spatially resolved model with cell population models describing later time NK cell responses. We have added a paragraph in the discussion section (appended below) to discuss this point.

In order to investigate the utility of our model to predict further downstream NK cell responses, we applied our model to predict lysis of target cells treated with DNA polymerase inhibitor aphidicolin in NK cell cytotoxic assays by Gasser et al.[62]. Gasser et al. [62] observed aphidicolin treatment moderately increases the expression of NKG2D ligands in mouse T-cell blast target cells and these target cells showed increased lysis compared to their untreated counterparts. An extension of our signaling model qualitatively predicts (S16 Fig) the differences in lysis of aphidicolin treated and untreated target cells for a range of effector cell:target cell ratios as reported by Gasser et al. [62]. This application could point to a potential way to combine early time signaling model with population kinetic models describing later time NK cell responses.

*2) Along the same lines, would the modeling have to be fitted each time for use in specific signaling systems or would their system be useful to describe microcluster formation in general? For example, the authors cite Steblyanko et. al. as a limitation of their work, however, would the in silico modeling be predictive of these results? If not, I would share Reviewer 1’s concerns about overfitting. Also, it would limit the impact of this type of in silico modeling.*

We thank the Reviewer for the comment. We think the issue of overfitting raised by the Reviewer#2 here and another reviewer (Reviewer #1in the original review) for the first submission arose from the lack of justification of the NKG2D signaling reactions used in the model. We have revised the description of the model in the main text to address this issue (also see our response for the next comment). The absence of the application of the model to describe another experiment also contributed to the issue of overfitting raised by Reviewer #1 in the first submission. We addressed this by using our model to describe NKG2D microcluster formation in confocal experiments carried out by Davis and colleagues reported in Brown et al. PLOS Biology 2011 (Ref. [7] in our manuscript) (S8 Fig).

The work by Steblyanko et al. considers cluster formation by activating CD16 receptors. CD16 in human NK cells are associated with CD3ζ and FcϵRIγ adaptors that can bind to several molecules of kinases such as Syk and Zap70 upon ITAM phosphorylation. Vav1 is a substrate for Syk family kinases, thus Vav1 phosphorylation due to CD16 signaling occurs via a different set of signaling reactions. Therefore, a direct application of our NKG2D signaling model could describe formation and centripetal movements of CD16 microclusters qualitatively, as the formation and movements of CD16 could arise from similar interplay between pVav1 kinetics and cytoskeletal reorganizations modeled here, however, the time scales of formation and centripetal movements of CD16 would depend on the time scales of pVav1 production. Since, the signaling reactions leading to pVav1 production are different between NKG2D and CD16, the spatiotemporal kinetics of NKG2D and CD16 are likely be quantitatively different. We have added a paragraph (appended below) in the Discussion section to addresses this point.

Therefore, we believe our model is able to describe a wide range of experimental scenarios for NKG2D signaling both quantitatively and qualitatively (see our response to comment #1 for Reviewer#2), and for other activating signaling receptors qualitatively.

Our NKG2D signaling model could describe spatiotemporal clustering by other activating NKRs such as CD16 qualitatively, as the formation and movements of another activating NKR microclusters could arise from similar interplay between pVav1 kinetics and cytoskeletal reorganizations modeled here, however, the time scales of formation and centripetal movements of microclusters of activating NKRs would depend on the time scales of pVav1 production. Since, the signaling reactions leading to pVav1 production are can be different between NKG2D and another activating NKR (e.g., CD16, KIR2DS1), the spatiotemporal kinetics of NKG2D and other activating NKRs are likely be quantitatively different.

*3) The other concern related to overfitting, their biochemical signaling processes are limited to a handful of papers. The models would rely heavily on the accuracy of the original paper findings. Can the authors validate their parameters across different publications/published results? Without external validation of the modeling, it’s difficult to evaluate the significance of the findings.*

The signaling reactions used in our model have been extensively investigated and validated in experiments labs of many investigators including Leibson, Billadeau, Long, Veillette, and others. We have expanded our modelling description (pages 4 - 5 in the revised manuscript) to include citations to these papers and further justify the signaling reactions we have used in our model. In addition, we have used our model to quantitatively describe microcluster formation by NKG2D in confocal experiments by Davis and colleagues (Ref. [7] in our manuscript) which was not used for model training (S8 Fig). Furthermore, we show in this revised version, that our model can qualitatively capture the NK cell response to populations of target cells expressing different amounts of NKG2D ligands. All the above, we believe, justify the construction of our signaling reactions, address overfitting issues, and the demonstrate the utility of the model in probing novel scenarios not considered in model training.

It will be difficult to validate the estimated parameters against published results because, 1) the available measurements are carried out in under different conditions (e.g., in vitro, truncated proteins) than that modeled here, 2) several of our estimated parameters are not well estimated and show large uncertainties. Therefore, we used available published values to set parameter ranges in our PSO estimation of parameters and used estimated parameters to generate predictions regarding NKG2D clustering and NK cell responses that are tested against experiments as we have responded above.

*4) During the discussion of microclusters, please cite these*

*papers:* [*doi.org/10.3389/fimmu.2012.00421*](https://urldefense.com/v3/__http:/doi.org/10.3389/fimmu.2012.00421__;!!ODYTt7Uqo6LDP14!1M2dhxKOeZGCseMfKP_STmK8y79oPv8W8RGvQdMgqWKO4umRaj0H9GcCrJLvtqtZniYPZ1tRKpZZ$)*, doi: 10.1038/nri2381.*

We have cited this paper in the revised manuscript.
